# Treatment-Related Predictive and Prognostic Factors in Trimodality Approach in Stage IIIA/N2 Non-Small Cell Lung Cancer

**DOI:** 10.3389/fonc.2018.00030

**Published:** 2018-02-20

**Authors:** Branislav Jeremić, Francesc Casas, Pavol Dubinsky, Antonio Gomez-Caamano, Nikola Čihorić, Gregory Videtic, Ivan Igrutinovic

**Affiliations:** ^1^BioIRC R&D Centre for Biomedical Research, Kragujevac, Serbia; ^2^University Clinic, Barcelona, Spain; ^3^University Hospital to East Slovakia Institute of Oncology, Kosice, Slovakia; ^4^University Hospital, Santiago de Compostela, Spain; ^5^Department of Radiation Oncology, Inselspital, Bern University Hospital, University of Bern, Bern, Switzerland; ^6^Cleveland Clinic, Cleveland, OH, United States; ^7^Faculty of Science, University of Kragujevac, Kragujevac, Serbia

**Keywords:** predictive factors, prognostic factors, stage IIIA/pN2, non-small cell lung cancer, trimodality therapy

## Abstract

While there are no established pretreatment predictive and prognostic factors in patients with stage IIIA/pN2 non-small cell lung cancer (NSCLC) indicating a benefit to surgery as a part of trimodality approach, little is known about treatment-related predictive and prognostic factors in this setting. A literature search was conducted to identify possible treatment-related predictive and prognostic factors for patients for whom trimodality approach was reported on. Overall survival was the primary endpoint of this study. Of 30 identified studies, there were two phase II studies, 5 “prospective” studies, and 23 retrospective studies. No study was found which specifically looked at treatment-related predictive factors of improved outcomes in trimodality treatment. Of potential treatment-related prognostic factors, the least frequently analyzed factors among 30 available studies were overall pathologic stage after preoperative treatment and UICC downstaging. Evaluation of treatment response before surgery and by pathologic tumor stage after induction therapy were analyzed in slightly more than 40% of studies and found not to influence survival. More frequently studied factors—resection status, degree of tumor regression, and pathologic nodal stage after induction therapy as well as the most frequently studied factor, the treatment (in almost 75% studies)—showed no discernible impact on survival, due to conflicting results. Currently, it is impossible to identify any treatment-related predictive or prognostic factors for selecting surgery in the treatment of patients with stage IIIA/pN2 NSCLC.

## Introduction

Stage III non-small cell lung cancer (NSCLC) is a heterogeneous disease presentation based on the range of patient and tumor characteristics included under its heading. Concurrent radio-chemotherapy (RT-CHT) is the standard treatment approach for locally advanced stage III NSCLC (1–3). In patients with stage IIIA/pN2 disease, thoracic oncologists have explored various means of optimizing treatment approach. Treatment regimens consisting of surgery alone or in combination with adjuvant CHT- and/or RT have been historically among the most common approaches used, with recent decades showing a trend for induction regimens followed by surgery. The latter have included induction CHT followed by surgery (4–7), and, in more recent years, induction RT-CHT followed by surgery (8–34). When tested in prospective randomized phase III studies, preoperative RT (PORT)-CHT (with or without preceding CHT) followed by surgery brought no improvement in overall survival (OS) or local control compared to definitive concurrent RT-CHT, and was associated also with an increase in treatment-related mortality (8, 10, 35, 36).

Notwithstanding this high level evidence, there exists reluctance among many clinicians to rule out the use of surgery in stage III NSCLC. For example, in an unplanned subgroup analysis of the Intergroup 0139 trial (10), it appeared that patients who underwent lobectomy as a component of their trimodality regimen had improved OS compared to the patients who received definitive RT-CHT. Given the controversial role of surgery in the management of this disease, looking at patient, tumor, and treatment variables from the range of studies in stage III NSCLC might then provide guidance as to which might favor including surgery as part of patient care.

Recently, a survey of the literature tested the role of surgical treatment in the care of stage III NSCLC patients (37). Important finding was that there were no pretreatment factors that identified any patient subgroup whose outcomes were improved by the addition of surgery. In addition, the majority of studies did not consistently evaluate prognostic factors that may have had an impact on treatment outcomes, but when done, none of the three most frequently analyzed factors (age, gender, histology) was found to clearly influence survival. From this, it was concluded that there were no specific pretreatment factors which favorably selected patients for surgery as a part of trimodality program (over definitive concurrent RT-CHT).

While little is known about treatment-related factors, the aim of the present study is to identify if potential treatment-related (i.e., after completion of induction therapy) predictive and prognostic factors exist that can help optimize the selection of patients for a trimodality approach for their stage IIIA/pN2 disease.

## Materials and Methods

References were identified through a literature search using PubMed, Google Scholar, EMBASE the terms “non-small cell lung cancer,” “surgery,” “radiotherapy,” “chemotherapy,” and “Stage IIIA” for the interval from 1990 until October 2017 as well as through searches of the references of the identified articles as well as of the authors’ own files. The year 1990 was selected as the starting point for the survey to mark for the contemporary era of RT delivery. Findings were restricted to English language publications in which potential treatment-related predictive and prognostic factors in the trimodality setting of concurrent RT-CHT (with or without preceding CHT) followed by surgery primarily focusing on Stage IIIA/pN2 (Stage III) were investigated. We principally sought out the highest level of evidence (from prospective randomized phase III trials). Upon finding none, the search was expanded to include prospective research (formal phase II or studies described only as “prospective”), and following that, all available retrospective studies using a trimodality approach meeting the criteria detailed above. Due to substantial heterogeneity in the sources, studies invariably included those with a mixture of patients (i.e., with both early and late stages NSCLC); they have also included patients treated with a variety of adjuvant therapies [e.g., PORT, or postoperative CHT (POCHT)]. Studies involving patients with superior sulcus/Pancoast/thoracic inlet tumors were excluded from the search. No study was excluded based on the form of induction CHT (drugs, regimens, administration, number of cycles), the form of RT (total doses delivered, once- or twice-daily fractionation, split- or continuous course), the type of surgery (pneumonectomy, bilobectomy, lobectomy) or adjuvant therapies (PORT or POCHT) employed. Any potential predictive or prognostic factor that was considered in a given study was included in the analysis.

Using Clark methodology (38, 39) *predictive factor* was defined as the one which defines the effect of treatment on the tumor, while a *prognostic factor* was defined as one which defines the independent impact of a given variable on the patient outcome. If one attempts to meaningfully use such predictors to guide the selection patients for trimodality approach, evaluation of clinically relevant endpoints (e.g., survival) should be used while the independent influence of these factors on a relevant endpoint (e.g., survival) distinguished from their ability to predict a differential clinical benefit from the specific treatment (e.g., PORT-CHT followed by surgery) (38, 39).

The purpose of this analysis is to determine if the survival benefit of patients treated surgically relative to patients treated with exclusive concurrent RT-CHT differs by any treatment-related characteristic, and thus establish the significance of a potential predictor. To establish the significance of such potential prognostic factors, it would be necessary to demonstrate its independent influence on treatment outcome. The current analysis was, therefore, restricted to studies that have provided such data and excluded studies that either did not discuss these or those in which they were described in univariate analyses only. Also, when the study using both univariate and multivariate analysis (MVA) disclosed factors that were not shown to influence survival on univariate analysis (and, therefore, subsequently, not entered into a multivariate model), such study was excluded from consideration. Finally, we excluded from consideration all studies that used multivariate analyses for subgroups of patients that were a part of the whole cohort of patients entering the published study.

In both prospective and retrospective series, multivariate analyses are used to investigate (treatment-related) prognostic factors, while the detection of treatment-related predictive factors requires more stringent criteria (38, 39). For the latter, one must determine if the survival benefit of patients on the investigational arm relative to patients on the control arm differ by treatment characteristic status. As an example, when survival by treatment arm for patients with documented mediastinal downstaging is compared to that for patients without this finding, one may find that the relative treatment benefit is similar. Then, looking at the survival results for all combinations of treatments and in whom documented mediastinal downstaging or not is determined, and if tests for interaction between mediastinal downstaging and treatment is not statistically significant, one may conclude that although mediastinal downstaging is a strong prognostic factor, it does not predict a differential survival benefit between the experimental treatment and the control treatment dependent on mediastinal downstaging.

Overall survival was the primary endpoint in this analysis and was provided in all identified studies. Loco-regional and distant metastasis-free survival as well as treatment-related toxicity, including serious adverse events, were not considered endpoints for this study, since they were absent in some of the identified studies. When presenting the study finding we used a qualitative approach (yes vs no) (Table 1). Tests of significance for a given study were not included in our summary table. Individual patient data was not sought for this analysis.

**Table 1:** Treatment characteristics as potential prognosticators.

Reference	Study	Response before surgery^a^	Treatment^a^ (all possible variables)	Resection status	Degree of tumor regression^a^	yp Stage	ypT^a^	ypN	UICC downstaging
Adelstein et al. (16)	Prospective phase II	No—clinical	Not evaluated	Not evaluated	Yes—pCR or pPR vs pNR	Not evaluated	Not evaluated	No—pN0	Not evaluated
Park et al. (40)		Not evaluated		No—R0	Not evaluated			Yes—pN0-1	
Choi et al. (11)	Prospective	No		Yes (CR—R0)				Yes—yp0 vs ypN1-2	
Thomas et al. (12)		Not evaluated		Yes (R0 vs R+)	Yes (>90 vs <90%)			Not evaluated	
Pezzetta et al. (22)			No—induction CHT vs induction RT-CHT	Not evaluated	Not evaluated			Yes—ypN0 vs ypN1-2	
Kim et al. (18)			Not evaluated		No—Necrosisproportion; no—viable tumor proportion		No—pT0-pT4		
Isobe et al. (41)		No (clinical response)	Not evaluated	No (resectability)	Yes	No	Not evaluated	No (pNo vs pN2-3)	
Cyjon et al (20).	Retrospective	No	No—number of CHT cycles	Yes—CR (R0)	Not evaluated	Not evaluated	Not evaluated	Not evaluated	
Sawabata et al. (21)		Not evaluated	Not evaluated	No—R0 vs R1			No—pT0-pT4	Yes -pN0-pN2 (single) vs pN2 (multiple)-N3	
Takeda et al. (13)		No	No	Not evaluated			Not evaluated	Not evaluated	
Caglar et al. (14)		Not evaluated	Yes—surgery; no—CHT						
Steger et al. (15)			No—Extent of surgery (pneumonectomy)		No—Regression score >IIb (<10% viable tumor tissue)		No—ypT downstaging	No	Yes—ypUICC
Li (23)		No (CR-PR vs SD-PD)	No—RT; no—POCHT	Yes—R0 vs R+	Yes—pCR or pPR vs pNR		Not evaluated	Yes—ypN0-1 vs ypN2 (mediastinal downstaging)	Not evaluated
Li J (24)		Not evaluated	Not evaluated					Not evaluated	
Weder et al (25)		No	No—type of surgery (pneumonectomy)		Not evaluated	No—ypStage	No—ypT	No—ypN	
Meacci et al. (26)			No—extent of surgery	Yes (R0 vs R1)		Not evaluated	Not evaluated	not evaluated	
Kim et al (27)		Not evaluated	Yes—extent of surgery (worse for pneumonectomy)	Not evaluated		No—yp0 (CR) vs yp (I + II + III)		No—ypN0 vs ypN1-2	
Shumway et al. (28)			No—RT, No—CHT; no—extent of surgery			Not evaluated		Not evaluated	
Shintani et al. (29)		No	Not evaluated					Yes—pN0-1 vs pN2(mediastinal downstaging)	
Steger et al. (30)		Not evaluated				No—ypT0N0M0	No	No	Yes—ypUICC
Gómez-Caro et al. (31)			Yes (trimodality superior to surgery only)			No—pStage (I–II/III–IV)	No—yp1-2 vs ypT3-4	Not evaluated	Not evaluated
Toyooka et al. (32)			Yes—(induction CRT superior to induction CT); no—extent of surgery		No—pCR (+ vs -)	Not evaluated	No—ypT	Yes—yp0-1 vs ypN2(mediastinal downstaging)	
Askoxylakis et al. (42)			No—induction CHT; no—non-CDDP		Not evaluated		Not evaluated	Not evaluated	
Lee et al. (43)			Yes—type of CHT; yes—type of surgery (worse for pneumonectomy); yes—PORT; POCHT	No—resection margin (negative vs close/positive)			No—ypT0 vs ypT1-4	Yes—ypN0 vs ypN+	
Renaud et al. (45)		No (pN downstaging after induction treatment)	No (type of surgery)	Not evaluated			No	Yes	
Lim et al (34)		No—clinical response; no—tumor size on post-induction treatment; yes—% change of target lesions on post-induction treatment	No—type of operation; no—adjuvant treatment	Not evaluated			No—total tumor size on surgical specimen; yes—viable tumor size on surgical specimen	No—pN0, pN1, pN2	
Shien et al. (46)		No (radiological response)	No (type of surgery)		No (pCR)		No (pT3 vs pTN0-2)	No (pN0-1 vs pN2-3)	
Darling et al. (47)		Not evaluated	Yes (surgery)		Not evaluated		Not evaluated	Not evaluated	
Kim et al. (44)		Not evaluated	Yes—type of surgery (worse for pneumonectomy)	Yes—close or positive margins			Yes—advanced pT	Yes—persistent N2	
Yang et al (48)			Yes (surgery)	Not evaluated			Not evaluated	Not evaluated	
Summary	Yes/no/not evaluated	1/13/18	10/19/10	8/4/18	5/5/21	0/5/25	2/11/18	11/8/11	2/0/28
INTERPRET		Unknown; possibly not	Possibly not	Unknown	Unknown	Unknown	Unknown; possibly not	Unknown	Unknown

*^a^More than one finding per study (the same for both RT, chemo, surgery)*.

*n.e., not evaluated; UICC, International Union Against Cancer; yp, pathological evaluation done after induction treatment (on operative specimen); pCR, pathological complete response; pPR, pathological partial response; pNR, pathological no response; R0, negative surgical margins; R1, microscopically positive resected tumor margins; R+, positive resected tumor margins; RT, radiotherapy; CHT, chemotherapy; PORT, postoperative RT; POCHT, postoperative CHT*.

## Results

Using Clark methodology (38, 39), we did not identify any study investigating treatment-related predictive factors for the potential superiority of surgical outcomes in patients with Stage IIIA (mostly pN2) NSCLC. A total of 30 studies which provided information on prognostic factors in trimodality studies using MVA (Cox proportional hazards model) were found for the interval of interest. Of these, there were two prospective phase II studies (16, 40), five prospective studies (11, 12, 18, 22, 41), and 23 retrospective studies (13–15, 20, 21, 23–32, 34, 42–48) (Table 1). Of these 30 studies, 17 were single arm studies, while the remaining 13 were two-arm studies. Of the latter, five studies compared concurrent RT-CHT vs a trimodality approach, one compared surgery alone with a trimodality approach, while the remaining seven studies compared induction CHT followed by surgery with induction RT-CHT followed by surgery. Of the total of 30 studies, 12 studies either allowed (in cases of high-risk features) or mandated (irrespective of risk features) the use of various forms of adjuvant or postoperative treatment. This included PORT alone, POCHT alone as well as postoperative RT-CHT. The total number of patients in these 30 studies was 3,397, of which 3,071 (90%) patients underwent surgical resection. Total of 14 studies included Stages IIIA and IIIB patients, three studies included patients with earlier (IB, II, and IIB) and/or higher (IV) stages. The 13 remaining studies defined staging through status of the nodes, of which the majority had N2 disease. All studies considered used OS as an endpoint.

A total of eight treatment-related variables were examined as potential prognosticators in these studies (Table 1). Of these, UICC downstaging and pathologic staging post-induction therapy (ypStaging) were the least frequently analyzed (in 2 and 5 out of 30 available studies, respectively) and, thus, cannot provide a measure of their influence on treatment outcome. Evaluation of treatment response before surgery (by clinical means) and pathologic tumor size after induction therapy (ypT) was looked at in slightly more than 40% of studies and found likely not to influence survival. Although done most frequently, investigations of the influence of treatment modalities, when given either alone or in various combinations, revealed no consistent result. In particular, there was an equal number of positive and negative studies investigating induction CHT vs induction RT-CHT, and which, between studies, showed either a positive or negative influence of the extent of surgery, in particular pneumonectomy (vs lesser operations). Of three clinically recognized important prognosticators, namely, surgical resection status (R0 vs R1 or R0 vs R+), degree of tumor regression after induction treatment as well as pathologic nodal status after induction (ypN) (in which there was great variability in reporting) analyses again revealed inconclusive results for the majority of eligible 30 studies (Table 1).

## Discussion

The present study looked at treatment-related factors that could aid in optimizing patient selection in the setting of trimodality treatment approach for Stage IIIA (primarily pN2) NSCLC. Similar to a recent study on pretreatment predictive and prognostic factors (37), we first noted inconsistent use of clinical definitions in this setting by clinicians. While we employed the definition of Clark (38, 39) to identify potential predictive factors, we found that that term was inaccurately used in most studies which then led to their incorrect inclusion in MVAs. In other words, MVAs reveal prognostic factors and not predictive ones.

Second, we found wide heterogeneity among studies with respect to diagnostic, staging, and treatment procedures, as well as different systems used to detect tumor response in surgical specimens. This led to significant inconsistencies in how potential treatment-related factors could be investigated. In particular, the majority of studies have used mediastinoscopy to prove pN2 while in some studies pathological mediastinal disease was found during surgery. It introduces potential bias in interpretation of the study results as patients with preoperatively confirmed N2 may have had worse prognosis than those with pN2 found at surgery and, possibly, could have different predictive and prognostic factors in the trimodality setting of Stage IIIA NSCLC.

Mindful of these shortcomings, we were unable to identify any prospective randomized study which investigated predictive factors in the setting of trimodality therapy, or the group of retrospective studies which compared bi- and trimodality approach. Third, we found that potential treatment-related prognostic factors were not consistently evaluated. Factors, such as post-induction therapy staging (ypStaging) or UICC downstaging, were looked at in less than 20% of all eligible studies; while factors, such as the degree of tumor regression, status of surgical margins, evaluation of response before surgery, or post-induction tumor status (ypT), were evaluated slightly more than 40% of all available studies. This limited our ability to detect the potential influence of any of these factors on treatment outcome. The factor more often investigated, post-induction nodal status (ypN), presented the same difficulty. No firm conclusions could be drawn possibly because of inconsistent definitions used for this across studies: some investigators used different comparators, either focusing on mediastinal nodal clearance (i.e., ypN0-1 vs ypN2), or only on ypN0 vs ypN+ or simply testing for the independent influence of a variable ypN (yes vs no).

The variable use of adjuvant treatments further contributed to the difficulty in assessing post-induction factors: while the majority of studies did not involve their use, for those that did, some allowed it in high-risk patients while others mandated their use, irrespective of surgical/pathological findings. It is possible that by allowing post-surgical therapy to be employed, the natural course of the disease treated with trimodality therapy was improved by, perhaps, improving both locoregional tumor control (with PORT) and/or microscopic tumor burden (with POCHT), and thus potentially achieving superior results when compared to those studies using no adjuvant treatment. Indeed, while resection status has long been considered as one of the major factors in predicting poor surgical outcomes, such that R0 is considered superior to any R+ status, we observed no such consistent association between studies and could not establish the importance of this factor.

Radiographic (clinical) response evaluations after induction therapy had a limited role in our analysis. In that regard, it is generally accepted that these are unreliable measures of the benefits of therapy as they are easily confounded. Contemporary studies that use novel approaches (e.g., PET-CT based response evaluations) and which could help in identifying such factors remain to be published. Future studies are required that incorporate this form of imaging which is then correlated with surgical/pathological findings.

The form of the surgical approach (all approaches considered) was also shown not to influence survival. The extent of surgery (pneumonectomy vs lesser operations) may simply be a surrogate for the bulk of the disease, so that the larger tumor burden by its nature carries the worse prognosis independent of surgical approach. A consistent definition of tumor burden prior to surgical intervention would clarify the impact of surgery in future studies where pneumonectomy is allowed since the decision of pneumonectomy vs lobectomy is also dependent on the location of the primary and nodal disease. This seems to be very important aspects, especially after Intergroup 0139 study results became available (10) showing worse survival for patients treated with trimodality approach using pneumonectomy vs lobectomy. Likewise, consistent stratification factors and trial eligibility criteria across studies and between research consortia would help better interpret conflicting results such as those in the histological domain which in EORTC study (8) favored squamous cell carcinoma while in Nordic study (36) favored adenocarcinoma.

The findings of the current study highlight our inability to use published study-derived treatment-related factors as either predictors or prognosticators to evaluate their impact in this clinical setting and to aid in the decision-making process regarding the appropriateness of surgery in stage III NSCLC. They follow our previous effort which focused on the pretreatment patient and tumor predictive and/or prognostic factors in this setting (37). Similar to it (37), although with slightly different number of included studies, the current one also could not identify such factors. This brings significant uncertainty in the process of decision-making before and after induction treatment was administered. It also reemphasizes the need for a more universal approach to defining and exploring treatment-related predictive and prognostic factor analysis in future studies, which needs to be coordinated across groups and countries. Possible avenues for clinical research in this setting would include more PRCTs with statistical analysis including predictive factor analysis being mandatory. In addition, a number of studies identified treatment-related factors that need to be further tested for its predictive and/or prognostic influence in this setting. This could be done as a preliminary step in the research scenario and, if repeatedly positive, across of various groups and institutions as to identify likely candidates for entering more patient and/or treatment-shaped trial milieu.

## Author Contributions

BJ: study design; literature search, data collection, manuscript draft, and final approval of the manuscript. FC, PD, AG-C, NC, GV, and II: literature search, data collection, manuscript draft, and final approval of the manuscript.

## Conflict of Interest Statement

The authors declare that the research was conducted in the absence of any commercial or financial relationships that could be construed as a potential conflict of interest.

## References

[1] Aupérin A Le Péchoux C Rolland E Curran WJ Furuse K Fournel P Meta-analysis of concomitant versus sequential radiochemotherapy in locally advanced non-small-cell lung cancer. J Clin Oncol (2010) 28:2181–90. 10.1200/JCO.2009.26.2543 20351327

[2] Liang H-Y Zhou H Li H-L Guan P Zhou BS Chemo-radiotherapy for advanced non-small cell lung cancer: concurrent or sequential? It’s no longer the question: a systematic review. Int J Cancer (2010) 127:718–28. 10.1002/ijc.25087 19957329

[3] O’Rourke N Roqué I Figuls M Farré Bernadó N Macbeth F Concurrent chemoradiotherapy in non-small cell lung cancer. Cochrane Database Syst Rev (2010) 2010:CD002140 10.1002/14651858.CD002140.pub3 PMC12584098 20556756

[4] Pass HI Pogrebniak HW Steinberg SM Mulshine J Minna J. Randomized trial of neoadjuvant therapy for lung cancer: interim analysis. Ann Thorac Surg (1992) 53:992–8. 10.1016/0003-4975(92)90373-C 1317697

[5] Rosell R Gomez-Codina J Camps C Maestre J Padille J Cantó A A randomized trial comparing preoperative chemotherapy plus surgery with surgery alone in patients with non-small-cell lung cancer. N Engl J Med (1994) 330:153–8. 10.1056/NEJM199401203300301 8043059

[6] Roth JA Fossella F Komaki R Ryan MB Putnam JB Jr Lee JS A randomized trial comparing perioperative chemotherapy and surgery with surgery alone in resectable stage IIIA non-small-cell lung cancer. J Natl Cancer Inst (1994) 86:673–80. 10.1093/jnci/86.9.673 8158698

[7] Nagai K Tsuchiya R Mori T Tada H Ichinose Y Koike T A randomized trial comparing induction chemotherapy followed by surgery with surgery alone for patients with stage IIIA N2 non-small cell lung cancer (JCOG 9209). J Thorac Cardiovasc Surg (2003) 125:254–60. 10.1067/mtc.2003.15 12579093

[8] van Meerbeeck JP Kramer GW van Schil PE Legrand C Smit EF Schramel F Randomized controlled trial of resection versus radiotherapy after induction chemotherapy in stage IIIA-N2 non-small-cell lung cancer. J Natl Cancer Inst (2007) 99:442–50. 10.1093/jnci/djk093 17374834

[9] Thomas M Rübe C Hoffknecht P Macha HN Freitag L Linder A Effect of preoperative chemoradiation in addition to preoperative chemotherapy: a randomised trial in stage III non-small-cell lung cancer. Lancet Oncol (2008) 9:636–48. 10.1016/S1470-2045(08)70156-6 18583190

[10] Albain KS Swann RS Rusch VW Turrisi AT III Shepherd FA Smith C Radiotherapy plus chemotherapy with or without surgical resection for stage III non-small-cell lung cancer: a phase III randomised controlled trial. Lancet (2009) 374:379–86. 10.1016/S0140-6736(09)60737-6 19632716 PMC4407808

[11] Choi NC Carey RW Daly W Mathisen D Wain J Wright C Potential impact on survival of improved tumor downstaging and resection rate by preoperative twice-daily radiation and concurrent chemotherapy in stage IIIA non-small-cell lung cancer. J Clin Oncol (1997) 15:712–22. 10.1200/JCO.1997.15.2.712 9053497

[12] Thomas MS Rübe C Semik M von Eiff M Freitag L Macha HN Impact of preoperative bimodality induction including twice-daily radiation on tumor regression and survival in stage III non–small-cell lung cancer. J Clin Oncol (1999) 17:1185–93. 10.1200/JCO.1999.17.4.1185 10561177

[13] Takeda S Maeda H Okada T Yamaguchi T Nakagawa M Yokota S Results of pulmonary resection following neoadjuvant therapy for locally advanced (IIIA—IIIB) lung cancer. Eur J Cardiothorac Surg (2006) 30:184–9. 10.1016/j.ejcts.2006.03.054 16730452

[14] Caglar HB Baldini EH Othus M Rabin MS Bueno R Sugarbaker DJ Outcomes of patients with stage III nonsmall cell lung cancer treated with chemotherapy and radiation with and without surgery. Cancer (2009) 115:4156–66. 10.1002/cncr.24492 19551884

[15] Steger V Walles T Kosan B Walker T Kyriss T Veit S Trimodal therapy for histologically proven N2/3 non–small cell lung cancer: mid-term results and indicators for survival. Ann Thorac Surg (2009) 87:1676–83. 10.1016/j.athoracsur.2009.03.068 19463576

[16] Adelstein DJ Rice TW Rybicki LA Greskovich JF Jr Ciezki JP Carroll MA Accelerated hyperfractionated radiation, concurrent paclitaxel/cisplatin chemotherapy and surgery for stage III non-small cell lung cancer. Lung Cancer (2002) 36:167–74. 10.1016/S0169-5002(01)00468-8 11955651

[17] Okada M Tsubota N Yoshimura M Miyamoto Y Matsuoka H. Induction therapy for non-small cell lung cancer with involved mediastinal nodes in multiple stations. Chest (2000) 118:123–8. 10.1378/chest.118.1.123 10893369

[18] Kim KJ Ahn YC Lim DH Han J Park K Park JO Analyses on prognostic factors following tri-modality therapy for stage IIIa non-small cell lung cancer. Lung Cancer (2007) 55:329–36. 10.1016/j.lungcan.2006.10.024 17157950

[19] Vora SA Daly BD Blaszkowsky L McGrath JJ Bankoff M Supran S High dose radiation therapy and chemotherapy as induction treatment for stage III nonsmall cell lung carcinoma. Cancer (2000) 89:1946–52. 10.1002/1097-0142(20001101)89:9<1946::AID-CNCR10>3.0.CO;2-1 11064351

[20] Cyjon A Nili M Fink F Kramer MR Fenig E Sandbank J Advanced non-small cell lung cancer: induction chemotherapy and chemoradiation before operation. Ann Thorac Surg (2002) 74:342–7. 10.1016/S0003-4975(02)03719-0 12173811

[21] Sawabata N Keller SM Matsumura A Kawashima O Hirono T Osaka Y The impact of residual multi-level N2 disease after induction therapy for non-small cell lung cancer. Lung Cancer (2003) 42:69–77. 10.1016/S0169-5002(03)00245-9 14512190

[22] Pezzetta E Stupp R Zouhair A Guillou L Taffé P von Briel C Comparison of neoadjuvant cisplatin-based chemotherapy versus radiochemotherapy followed by resection for stage III (N2) NSCLC. Eur J Cardiothorac Surg (2005) 27:1092–8. 10.1016/j.ejcts.2005.02.035 15896624

[23] Li J Dai C-H Shi S-B Chen P Yu LC Wu JR Prognostic factors and long term results of neoadjuvant therapy followed by surgery in stage IIIA N2 non-small cell lung cancer patients. Ann Thorac Med (2009) 4:201–7. 10.4103/1817-1737.56010 19881166 PMC2801045

[24] Li J Dai C-H Yu L-C Chen P Li XQ Shi SB Results of trimodality therapy in patients with stage IIIA (N2-bulky) and stage IIIB non-small-cell lung cancer. Clin Lung Cancer (2009) 10:353–9. 10.3816/CLC.2009.n.048 19808194

[25] Weder W Collaud S Eberhardt WE Hillinger S Welter S Stahel R Pneumonectomy is a valuable treatment option after neoadjuvant therapy for stage III non-small-cell lung cancer. J Thorac Cardiovasc Surg (2010) 139:1424–30. 10.1016/j.jtcvs.2010.02.039 20416887

[26] Meacci E Cesario A Cusumano G Lococo F D’Angelillo R Dall’armi V Surgery for patients with persistent pathological N2 IIIA stage in non-small-cell lung cancer after induction radio-chemotherapy: the microscopic seed of doubt. Eur J Cardiothorac Surg (2011) 40:656–63. 10.1016/j.ejcts.2010.12.062 21402479

[27] Kim AW Liptay MJ Bonomi P Warren WH Basu S Farlow EC Neoadjuvant chemoradiation for clinically advanced non-small cell lung cancer: an analysis of 233 patients. Ann Thorac Surg (2011) 92:233–43. 10.1016/j.athoracsur.2011.03.001 21620372

[28] Shumway D Corbin K Salgia R Hoffman P Villaflor V Malik RM Pathologic response rates following definitive dose image-guided chemoradiotherapy and resection for locally advanced non-small cell lung cancer. Lung Cancer (2011) 74:446–50. 10.1016/j.lungcan.2011.05.003 21676484

[29] Shintani Y Funakoshi Y Inoue M Takeuchi Y Okumura M Maeda H Pathological status of mediastinal lymph nodes after preoperative concurrent chemoradiotherapy determines prognosis in patients with non-small cell lung cancer. Ann Thorac Cardiovasc Surg (2012) 18:530–5. 10.5761/atcs.oa.11.01811 22785450

[30] Steger V Spengler W Hetzel J Veit S Walker T Mustafi M Pneumonectomy: calculable or non-tolerable risk factor in trimodal therapy for Stage III non-small-cell lung cancer. Eur J Cardiothorac Surg (2012) 41:880–5. 10.1093/ejcts/ezr160 22233799

[31] Gómez-Caro A Boada M Reguart N Viñolas N Casas F Molins L. Sleeve lobectomy after induction chemoradiotherapy. Eur J Cardiothorac Surg (2012) 41:1052–8. 10.1093/ejcts/ezr184 22223693

[32] Toyooka S Kiura K Shien K Katsui K Hotta K Kanazawa S Induction chemoradiotherapy is superior to induction chemotherapy for the survival of non-small-cell lung cancer patients with pathological mediastinal lymph node metastasis. Interact Cardiovasc Thorac Surg (2012) 15:954–60. 10.1093/icvts/ivs412 22976995 PMC3501315

[33] Margaritora S Cesario A Cusumano G Dall’armi V Porziella V Meacci E Pneumonectomy with and without induction chemo-radiotherapy for non-small cell lung cancer: short and long-term results from a single centre. Eur Rev Med Pharmac Sci (2013) 17:29–40. 23329521

[34] Lim H Lee HY Lee KS Han J Kwon OJ Park K Predictive factors for survival in stage IIIA N2 NSCLC patients treated with neoadjuvant CCRT followed by surgery. Cancer Chemother Pharmacol (2015) 75:77–85. 10.1007/s00280-014-2619-1 25374409

[35] Eberhardt WE Pöttgen C Gauler TC Friedel G Veit S Heinrich V Phase III study of surgery versus definitive concurrent chemoradiotherapy boost in patients with resectable stage IIIA(N2) and selected IIIB non-small-cell lung cancer after induction chemotherapy and concurrent chemoradiotherapy (ESPATUE). J Clin Oncol (2015) 33:4194–201. 10.1200/JCO.2015.62.6812 26527789

[36] Sorensen JB Riska H Ravn J Palshof T Sundstrom S Bergman B Scandinavian phase III trial of neoadjuvant chemotherapy in NSCLC stages IB-IIIA/T3. J Clin Oncol (2013) 31(Suppl):abstr7504 10.1200/jco.2005.23.16_suppl.7146

[37] Jeremic B Casas F Dubinsky P Gomez-Caamano A Čihorić N Videtic G Surgery in Stage IIIA nonsmall cell lung cancer: lack of predictive and prognostic factors identifying any patient subgroup benefiting from it. Clin Lung Cancer (2016) 17:107–12. 10.1016/j.cllc.2015.11.001 26683387

[38] Clark GM. Prognostic factors versus predictive factors: examples from a clinical trial of erlotinib. Mol Oncol (2008) 1:406–12. 10.1016/j.molonc.2007.12.001 19383314 PMC5543832

[39] Clark GM Zborowski DM Culbertson JL Whitehead M Savoie M Seymour L Clinical utility of epidermal growth factor receptor expression for selecting patients with advanced non-small cell lung cancer for treatment with erlotinib. J Thorac Oncol (2006) 1:837–46. 10.1016/S1556-0864(15)30414-7 17409968

[40] Park B-B Park JO Kim H Ahn YC Choi YS Kim K Is trimodality approach better then bimodality in stage IIIA, N2 positive non-small cell lung cancer? Lung Cancer (2006) 53:323–30. 10.1016/j.lungcan.2006.05.024 16844258

[41] Isobe K Hata Y Sakaguchi S Sato F Takahashi S Sato K Pathological response and prognosis of stage III non-small cell lung cancer patients treated with induction chemoradiation. Asia Pac J Clin Oncol (2012) 8:260–6. 10.1111/j.1743-7563.2012.01529.x 22897895

[42] Askoxylakis V Tanner J Kappes J Hoffmann H Nicolay NH Rief H Trimodal therapy for stage III-N2 non-small-cell lung carcinoma: a single center retrospective analysis. BMC Cancer (2014) 14:572. 10.1186/1471-2407-14-572 25104240 PMC4137085

[43] Lee H Ahn YC Pyo H Kim B Oh D Nam H Pretreatment clinical mediastinal nodal bulk and extent do not influence survival in N2-positive stage IIIA non-small cell lung cancer patients treated with trimodality therapy. Ann Surg Oncol (2014) 21:2083–90. 10.1245/s10434-014-3540-x 24522994

[44] Kim HK Cho JH Choi YS Zo JI Shim YM Park K Outcomes of neoadjuvant concurrent chemoradiotherapy followed by surgery for non-small-cell lung cancer with N2 disease. Lung Cancer (2016) 96:56–62. 10.1016/j.lungcan.2016.03.016 27133751

[45] Renaud S Falcoz PE Olland A Reeb J Santelmo N Massard G Mediastinal downstaging after induction treatment is not a significant prognostic factor to select patients who would benefit from surgery: the clinical value of the lymph node ratio. Interact Cardiovasc Thorac Surg (2015) 20:222–7. 10.1093/icvts/ivu378 25413781

[46] Shien K Toyooka S Soh J Hotta K Katsui K Oto T Lower lobe origin is a poor prognostic factor in locally advanced non-small-cell lung cancer patients treated with induction chemoradiotherapy. Mol Clin Oncol (2015) 3:706–12. 10.3892/mco.2015.509 26137291 PMC4471621

[47] Darling GE Li F Patsios D Massey C Wallis AG Coate L Neoadjuvant chemoradiation and surgery improves survival outcomescompared with definitive chemoradiation in the treatment of stage IIIA N2 non-small-cell lung cancer. Eur J Cardiothorac Surg (2015) 48:684–90. 10.1093/ejcts/ezu504 25567960

[48] Yang KL Chang YC Ko HL Chi MS Wang HE Hsu PS Optimizing survival of patients with marginally operable stage IIIA non-small-cell lung cancer receiving chemoradiotherapy with or without surgery. Clin Lung Cancer (2016) 17:550–7. 10.1016/j.cllc.2016.05.013 27378175

